# Femtosecond Autocorrelation of Localized Surface Plasmons

**DOI:** 10.3390/nano13091513

**Published:** 2023-04-28

**Authors:** Ruizhi Yi, Wenwen Wu, Xinping Zhang

**Affiliations:** Institute of Information Photonics Technology, Beijing University of Technology, Beijing 100124, China

**Keywords:** localized surface plasmons, autocorrelation, femtosecond laser pulses, decay dynamics of collective electron oscillation, solution processed gold nanoparticles

## Abstract

Plasmon electronic dephasing lifetime is one of the most important characteristics of localized surface plasmons, which is crucial both for understanding the related photophysics and for their applications in photonic and optoelectronic devices. This lifetime is generally shorter than 100 fs and measured using the femtosecond pump–probe technique, which requires femtosecond laser amplifiers delivering pulses with a duration even as short as 10 fs. This implies a large-scale laser system with complicated pulse compression schemes, introducing high-cost and technological challenges. Meanwhile, the strong optical pulse from an amplifier induces more thermal-related effects, disturbing the precise resolution of the pure electronic dephasing lifetime. In this work, we use a simple autocorrelator design and integrate it with the sample of plasmonic nanostructures, where a femtosecond laser oscillator supplies the incident pulses for autocorrelation measurements. Thus, the measured autocorrelation trace carries the optical modulation on the incident pulses. The dephasing lifetime can be thus determined by a comparison between the theoretical fittings to the autocorrelation traces with and without the plasmonic modulation. The measured timescale for the autocorrelation modulation is an indirect determination of the plasmonic dephasing lifetime. This supplies a simple, rapid, and low-cost method for quantitative characterization of the ultrafast optical response of localized surface plasmons.

## 1. Introduction

Localized surface plasmons (LSPs) are essentially understood as collective oscillations of free electrons in nanostructured metals [1,2,3] and other conductive materials [4,5,6], which have been extensively investigated and applied in optoelectronic devices [7,8,9,10,11,12] and sensors [13,14,15,16]. Local-field and light-scattering enhancement by LSPs have been utilized in photovoltaic diodes [7,8], light-emitting devices [9,10], and various type of lasers [11,12]. Further applications of LSPs may involve optical logic circuits [17,18] and optical switching devices, which are particularly important for optical communication or optical computation techniques. Ultrafast plasmonic optical switching devices have been reported in a large variety of designs [19,20,21], which are based on the ultrafast modulation of electronic dynamics. In principle, the dephasing of the plasmonic electron oscillation takes less than 100 fs. However, due to the interaction with phonons, the plasmonic response of metallic nanostructures may have a lifetime, even longer than picoseconds. Moreover, inter-band transition processes [21] may be involved in the plasmonic response and the electronic dynamics will be strongly modulated, so that the corresponding lifetime of the plasmonic signal will be largely extended. Furthermore, an interference effect has been observed between the photon–electron interactions in metallic nanostructures [22], which will further broaden the transient optical spectroscopic response signals.

Therefore, the ultrafast optical response is one of the important characteristics of localized surface plasmons, which lays the basis for the applications of nanostructured metals in both photonic and optoelectronic devices. Dephasing lifetimes of a few femtoseconds have been measured for a single metallic nanostructure [23] and for colloidal gold nanoparticles [24] using interferometric frequency resolved optical gating and persistent spectral hole burning, respectively. The determination of the plasmonic dephasing lifetime is thus very important for understanding the responsible photophysics and for realizing related applications. Various methods have been reported for the measurement of the plasmonic dephasing lifetime, where metallic nanoparticles [25,26,27], nanorods [28], and waveguide metallic photonic crystals [29] have been investigated. Dependence of the dephasing lifetime on the diameter of gold nanoparticles was also evaluated [30]. Investigations using time-resolved photoemission electron microscopy (PEEM) revealed that the optical response function of localized surface plasmons in gold nanoparticles can be modeled from the interferometric autocorrelation signals using the using single and coupled Lorentzian oscillator models [31]. Femtosecond pump–probes are the most commonly employed technique for measurements of the plasmonic response time, in particular in its modulation of the optoelectronic processes in nanostructured metal/semiconductor systems [32]. However, such a system generally consists of a femtosecond laser amplifier with low repetition rates and large pulse energies, as well as a large-scale pump–probe system, which is not only very expensive, but also occupies a large space and requires very high laboratory conditions. Supercontinuum generation with a large bandwidth is also required for supplying the probe pulses. Complicated optics, computer-control systems with elaborately designed hard- and soft-wares, and long scanning times for data acquisition are required for such techniques. Although such an advanced system may supply much more detailed and multifold information about the optical response of the plasmonic devices, it is not a reasonable approach to simply characterize the plasmonic lifetime as simple parameter. Additionally, the pulse energy from the amplifiers is generally in the scale of micro- or milli-joules. Such strong pulses will inevitably induce phonon-related effects, extending the tail of the electronic dynamics curve or even submerging the true electronic dynamics.

Therefore, simple, convenient, and more straightforward approaches are expected for the rapid evaluation on the plasmon electronic response lifetime. In this work, we report a simple rotator autocorrelator scheme with the plasmonic nanostructures incorporated as part of the rotator for optical path length variation, so that the autocorrelation trace carries the plasmonic modulation. Thus, comparison between the autocorrelation traces with and without the plasmonic modulation is used to resolve the electronic dephasing lifetime, where fitting to the measurement data using the damped simple harmonic oscillator model enabled extraction of the plasmonic response lifetime.

## 2. Design of the Plasmonic Autocorrelation Measurement Scheme

**Figure 1:**
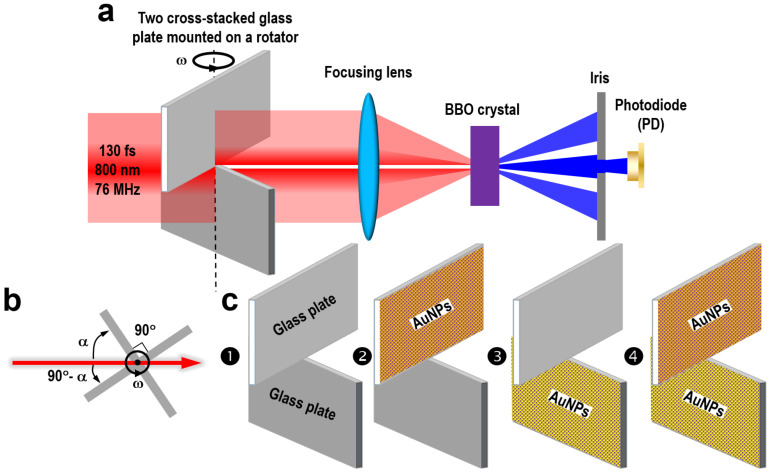
Experimental setup and basic principles for the autocorrelation measurement on localized surface plasmons (LSPs). (**a**) Autocorrelation measurement system using cross-stacked, delay-line rotator with transient SFG detection. (**b**) Basic principles for the cross-stacked, rotating delay line. (**c**) Designs of the LSP schemes of randomly distributed gold nanoparticles (AuNPs) for autocorrelation measurements.

Figure 1 shows the design of the experimental setup for the measurement of plasmonic autocorrelation. Laser pulses centered at 800 nm with a pulse length of 130 fs and a repetition rate of 76 MHz from a Ti:sapphire laser oscillator were sent to the autocorrelator after being re-collimated with a beam diameter of about 5 mm, as shown in Figure 1a. The rotator of the autocorrelator consisted of two cross-stacked glass plates with an included angle of 90°, which were mounted onto the shaft of a direct-current (DC) motor to constitute a rotator, as illustrated in both Figure 1a,b. The incident laser beam was arranged such that it was divided into two beams evenly by the two glass plates, as shown in Figure 1a, which pass through the upper and the lower plate, respectively. They were then focused into a barium boron oxide (BBO) crystal to produce second-harmonic generation (SHG).

In addition to their respective SHG signals, there will be a sum-frequency generation (SFG) signal in the bisecting direction, which is still the SHG of the incident laser pulses. As the two glass plate are rotating together, the laser pulses carried by the two beams will experience different time delays, because the laser pulses passing through different thickness of the glass plate for the upper and lower laser beams at *α* ≠ 45°. Thus, the rotator acts as a delay line between the two laser pulses, as the DC motor rotates continuously and smoothly. Measurement of the SFG signal intensity by a photodiode (PD) as a function of the delay between the two pulses produces the autocorrelation trace of the incident femtosecond pulse. Calibration is needed to convert the rotation angle of the autocorrelator to the time delay between the two split pulses, which is depicted as mode details in the following derivations.

**Figure 2:**
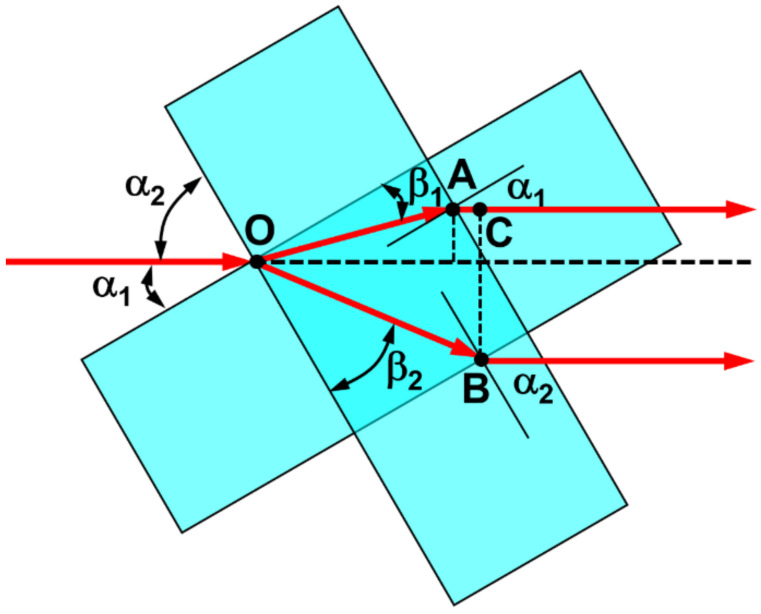
Geometric illustration of the delay between the two split pulses for autocorrelation measurement.

For understanding in details the basic principles of the autocorrelation measurement and for deriving the time delay between the two split pulses, we make a drawing of the geometric structures of the rotator consisting two cross-stacked glass plates, as shown in Figure 2. For the two glass plates, the laser pulse is assumed to be incident at angles of *α*_1_ and *α*_2_ onto the two glass plates, respectively. The corresponding refractions are at *β*_1_ and *β*_2_ into the glass plates, respectively, where $\beta_{1}=\sin^{-1}\left(\frac{\sin\alpha_{1}}{n}\right)$ and $\beta_{2}=\sin^{-1}\left(\frac{\sin\alpha_{2}}{n}\right)$ with *n* denoting the refractive index of the glass plate. Apparently, the delay between the pulses can be calculated by:

(1) $$\tau =\bar{OB}-\bar{OA}-\bar{AC}$$

According to Figure 2, we have:

(2) $$\bar{OB}=\frac{nd}{\cos\left(\beta_{2}\right)}=\frac{nd}{\sqrt{1-\frac{\sin^{2}\alpha_{2}}{n^{2}}}},$$

(3) $$\bar{OA}=\frac{nd}{\cos\left(\beta_{1}\right)}=\frac{nd}{\sqrt{1-\frac{\sin^{2}\alpha_{1}}{n^{2}}}},$$

(4) $$\begin{array}{cc} \bar{AC} & =\bar{OB}\cdot \cos\left(\alpha_{2}-\beta_{2}\right)-\bar{OA}\cdot \cos\left(\alpha_{1}-\beta_{1}\right)\\ & =\frac{nd\cos\left[\alpha_{2}-\sin^{-1}\left(\frac{{\sin\alpha }_{2}}{n}\right)\right]}{\sqrt{1-\frac{\sin^{2}\alpha_{2}}{n^{2}}}}-\frac{nd\cos\left[\alpha_{1}-\sin^{-1}\left(\frac{\sin\alpha_{1}}{n}\right)\right]}{\sqrt{1-\frac{\sin^{2}\alpha_{1}}{n^{2}}}}, \end{array}$$

where *d* is the thickness of the glass plates.

Considering that *α*_1_ + *α*_2_ = 90°, Equations (2) and (4) can be rewritten as:

(5) $$\bar{OB}=\frac{nd}{\cos\left(\beta_{2}\right)}=\frac{nd}{\sqrt{1-\frac{\cos^{2}\alpha_{1}}{n^{2}}}},$$

(6) $$\bar{AC}=\frac{nd\sin\left[\alpha_{1}+{\;\sin}^{-1}\left(\frac{{\cos\alpha }_{1}}{n}\right)\right]}{\sqrt{1-\frac{\cos^{2}\alpha_{1}}{n^{2}}}}-\frac{nd\cos\left[\alpha_{1}-\sin^{-1}\left(\frac{\sin\alpha_{1}}{n}\right)\right]}{\sqrt{1-\frac{\sin^{2}\alpha_{1}}{n^{2}}}}.$$

In fact, *α*_1_ is the rotation angle, which is a function of the rotation speed and the variation of time. Thus, we may assume *α*_1_ = *α* = *ωt*, where the rotation angle *α* and the angular rotation speed *ω* have been defined in Figure 1.

Therefore, we have the time delay modified as follows:

(7) $$\begin{array}{ccc} \tau \left(t\right) & =\frac{\bar{OB}-\bar{OA}-\bar{AC}}{c} & \\ & \;=\frac{nd}{c\sqrt{1-\frac{\cos^{2}\left(\omega t\right)}{n^{2}}}} & -\frac{nd}{c\sqrt{1-\frac{\sin^{2}\left(\omega t\right)}{n^{2}}}}\\ & - & \left\{\frac{nd\sin\left[\omega t+{\;\sin}^{-1}\left(\frac{\cos\left(\omega t\right)}{n}\right)\right]}{c\sqrt{1-\frac{\cos^{2}\left(\omega t\right)}{n^{2}}}}-\frac{nd\cos\left[\omega t-\sin^{-1}\left(\frac{\sin\left(\omega t\right)}{n}\right)\right]}{c\sqrt{1-\frac{\sin^{2}\left(\omega t\right)}{n^{2}}}}\right\}, \end{array}$$

where *c* is the velocity of light.

In fact, Equation (7) can be further simplified as:

(8) $$\tau \left(t\right)=\frac{n^{2}d}{c}\left\{\frac{1-\sin\left[\omega t+\sin^{-1}\left(\frac{\cos\left(\omega t\right)}{n}\right)\right]}{\sqrt{n^{2}-\cos^{2}\left(\omega t\right)}}-\frac{1-\cos\left[\omega t-\sin^{-1}\left(\frac{\sin\left(\omega t\right)}{n}\right)\right]}{\sqrt{n^{2}-\sin^{2}\left(\omega t\right)}}\right\},$$

However, the overlap between the two femtosecond pulses passing through the top and bottom plates takes place only within a very small range of the change of *α* deviating from 45°, where we have τ = 0 at *α* = *ωt* = 45°. This implies an approximation of $\bar{AC}\approx 0$. Thus, Equation (8) can be approximated as:

(9) $$\tau \left(t\right)\approx \frac{n^{2}d}{c}\left[\frac{1}{\sqrt{n^{2}-\cos^{2}\left(\omega t\right)}}-\frac{1}{\sqrt{n^{2}-\sin^{2}\left(\omega t\right)}}\right].$$

For the measurement of the LSP lifetime using intensity autocorrelation, we simply need to replace one or two of the glass plates with one coated with gold nanoparticles (AuNPs). Figure 1c➊ corresponds to the scheme without plasmonic modulation, and Figure 1c➋, ➌, and ➍ show the configurations of the AuNP-coated glass plate mounted on the top, the bottom, and on both locations, respectively. The LSP lifetime can be determined by the comparison between autocorrelation signals for the schemes with (➋, ➌, ➍) and without (➊) AuNPs. Apparently, the same LSP lifetime can be justified from schemes ➋, ➌, and ➍, if the AuNPs are fabricated under the same conditions for both the upper and lower plates. However, due to the different plasmonic modulations of the two pulsed laser beams, different autocorrelation signals were obtained for schemes ➋, ➌, and ➍, as will be demonstrated in the experimental results in Section 4.

**Figure 3:**
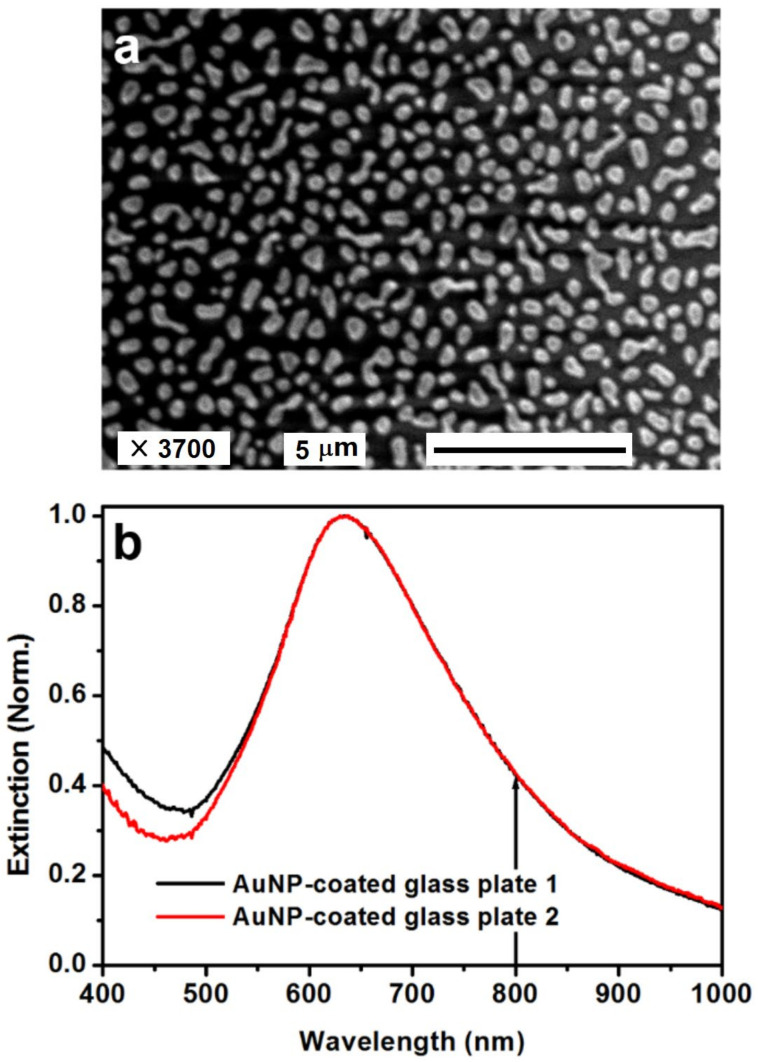
(**a**) SEM image of the AuNPs. (**b**) Optical extinction spectra measured from the two AuNP-coated glass plates.

In Figure 3, we show the microscopic and spectroscopic characterization of the randomly distributed AuNPs on the glass plates. Colloidal AuNPs in xylene with a concentration of 100 mg/mL was first spin-coated on the glass plate with a speed of 2000 rpm before the sample was annealed at 400 °C for 20 min in a Muffle furnace. The AuNPs were chemically synthesized, and were covered with ligands for good dispersity in xylene. Figure 3a shows the scanning electron microscopic (SEM) image of the annealed AuNPs. Since the AuNP-coated glass plates were fabricated using identical methods and parameters, only one SEM image is presented in Figure 3a. Due to the non-conductivity of the glass plate, the SEM image was not clear enough, influencing the precise determination of the size of the AuNPs. Although the AuNPs had a large distribution range for their sizes and shapes, a rough evaluation justified a mean diameter of about 430 nm. Figure 3b shows the optical extinction spectra measured from the two AuNP-coated glass plates which were employed in the scheme ➍. Clearly, the two spectra were basically identical to each other except for some discrepancies at wavelengths shorter than 550 nm. Both spectra peaked at about 633 nm, denoting the center resonance wavelength of localized surface plasmons. The bandwidth of these two spectra was measured to be about 190 nm at FWHM. Although the center wavelength of the femtosecond laser pulses, which was located at about 800 nm, was about 167 nm away from the plasmon resonance peak, it was still within the resonance spectrum, as shown in Figure 3b by an upward arrow, implying a reasonable experimental configuration.

## 3. Modeling of Plasmonic Autocorrelation

According to the design of the autocorrelation scheme in Figure 1, we are measuring an intensity autocorrelation function. In both the measurements and the simulations, we assume a pulse length of *τ_P_* = 130 fs for the incident laser pulses and define a plasmon electronic dephasing lifetime of *τ_LSP_*, a full width at half maximum (FWHM) of the autocorrelation trace of Δ*τ_AC_*. If assuming a Gaussian shape for the incident laser pulses, we may express the pulse intensity as:

(10) $$I\left(t\right)=Ae^{-4ln2{\left(\frac{t}{\tau_{P}}\right)}^{2}},$$

where *A* is a constant, denoting the peak intensity of the pulse.

Before the experimental investigation, we formulated the modeling of the autocorrelation function as:

(11) $$I_{AC}\left(\tau \right)=\int_{-\infty }^{\infty }I_{1}\left(t\right)\cdot I_{2}\left(t-\tau \right)dt,$$

where *I*_1_ and *I*_2_ are the pulse intensities passing through the upper and lower glass plates of the rotator, *I_A__C_*(*τ*) is the function of the autocorrelation trace, and *τ* is the delay between the upper and lower pulse, as defined in (9).

In consideration of the modulation by localized surface plasmons, we need to incorporate the plasmonic response function [33], which is based on the damped simple harmonic oscillator model:

(12) $$R\left(\omega \right)=\frac{1}{\omega_{r}^{2}+2\gamma i\omega -\omega^{2}},$$

where *ω*_r_ is the plasmonic resonance frequency and *γ* is the damping coefficient. Fourier transformation (FT) of Equation (12) produces the time-dependent response function,

(13) $$R\left(t\right)=te^{-a\gamma t}u\left(t\right)=te^{-\frac{at}{2\tau_{LSP}}}u\left(t\right),$$

where *a* is a constant coefficient produced during FT and *u(t)* is a unit step function.

The plasmonically modulated optical pulse for scheme ➋ can be expressed as:

(14) $$I_{P1}\left(t\right)=\int_{-\infty }^{\infty }I_{1}\left(\delta \right)\cdot R\left(t-\delta \right)d\delta ,$$

or for scheme ➌ as:

(15) $$I_{P2}\left(t\right)=\int_{-\infty }^{\infty }I_{2}\left(\delta \right)\cdot R\left(t-\delta \right)d\delta .$$

Thus, we formulate the autocorrelation trace for scheme ➋ as:

(16) $$I_{AC}\left(\tau \right)=\int_{-\infty }^{\infty }I_{P1}\left(t\right)\cdot I_{2}\left(t-\tau \right)dt,$$

or for scheme ➌ as:

(17) $$I_{AC}\left(\tau \right)=\int_{-\infty }^{\infty }I_{P2}\left(t\right)\cdot I_{1}\left(t-\tau \right)dt,$$

Apparently, Equations (16) and (17) are equivalent to each other if *I*_1_*(t)* and *I*_2_*(t)* have the same expression.

As for scheme ➍, both pulses passing through the upper and lower glass plates are plasmonically modulated, producing an autocorrelation trace formulated as:

(18) $$I_{AC}\left(\tau \right)=\int_{-\infty }^{\infty }I_{P1}\left(t\right)\cdot I_{P2}\left(t-\tau \right)dt.$$

**Figure 4:**
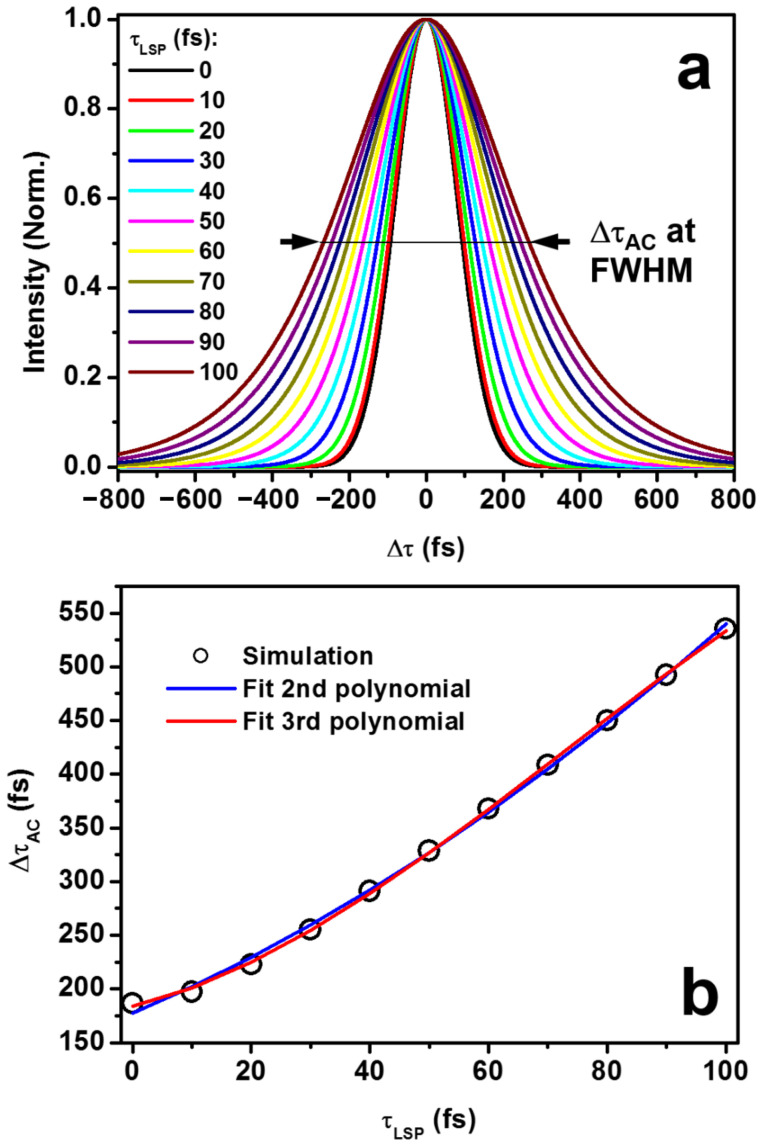
(**a**) Simulation results of the autocorrelation trace for different plasmonic dephasing lifetimes (*τ_LSP_*). (**b**) Plot of the autocorrelation signal width at FWHM as a function of *τ_LSP_*.

Using Equations (16)–(18), we may calculate the autocorrelation traces for different schemes of the design. Figure 4 shows the simulation results of the autocorrelation signals using the above model. In Figure 4a, we show the calculated autocorrelation traces with different plasmonic dephasing lifetime (*τ_LSP_*). *τ_LSP_* = 0 corresponds to the case of two bland glass plates without AuNPs in the design of the autocorrelator, corresponding to scheme ➊ in Figure 1c. For *τ_LSP_* ≠ 0, one of the glass plates is coated with AuNPs, corresponding to the scheme ➋ or ➌ in Figure 1c. As the value of *τ_LSP_* is increased from 0 to 100 fs, which is reasonable for the electronic dephasing in plasmonic nanostructures, the FWHM value of the autocorrelation trace (Δ*τ_AC_*) increased rapidly from that of the incident laser pulse of 183.8 fs to 535.4 fs. This implies a large broadening of the laser pulses due to the interaction with the plasmonic electrons.

However, the relationship between Δ*τ_AC_* and *τ_LSP_* is not a linear function, as shown in Figure 4b, which is a plot of Δ*τ_AC_* as a function of *τ_LSP_* (empty circles). The blue and red curves are fittings using 2nd- and 3rd-order polynomials, respectively. The 3rd-order polynomial shows a better fit than the 2nd-order with the simulation data. Therefore, larger values of *τ_LSP_* induced much stronger broadening effects on the incident light pulses. The nonlinear dependence can also be inferred from the theoretical model in Equations (13) and (14). Apparently, the 3rd-order polynomial is not a precise relationship; however, for *τ_P_* = 130 fs and *τ_LSP_* < 100, this relationship basically holds. The theoretical results in Figure 4 not only demonstrate broadening modulation of the autocorrelation signal by localized surface plasmons, but also verified that the modeling using Equations (9)–(13) may supply an effective tool to evaluate the plasmonic dephasing lifetime through fitting the measured autocorrelation trace.

## 4. Plasmonic Autocorrelation Measurements on Gold Nanoparticles

**Figure 5:**
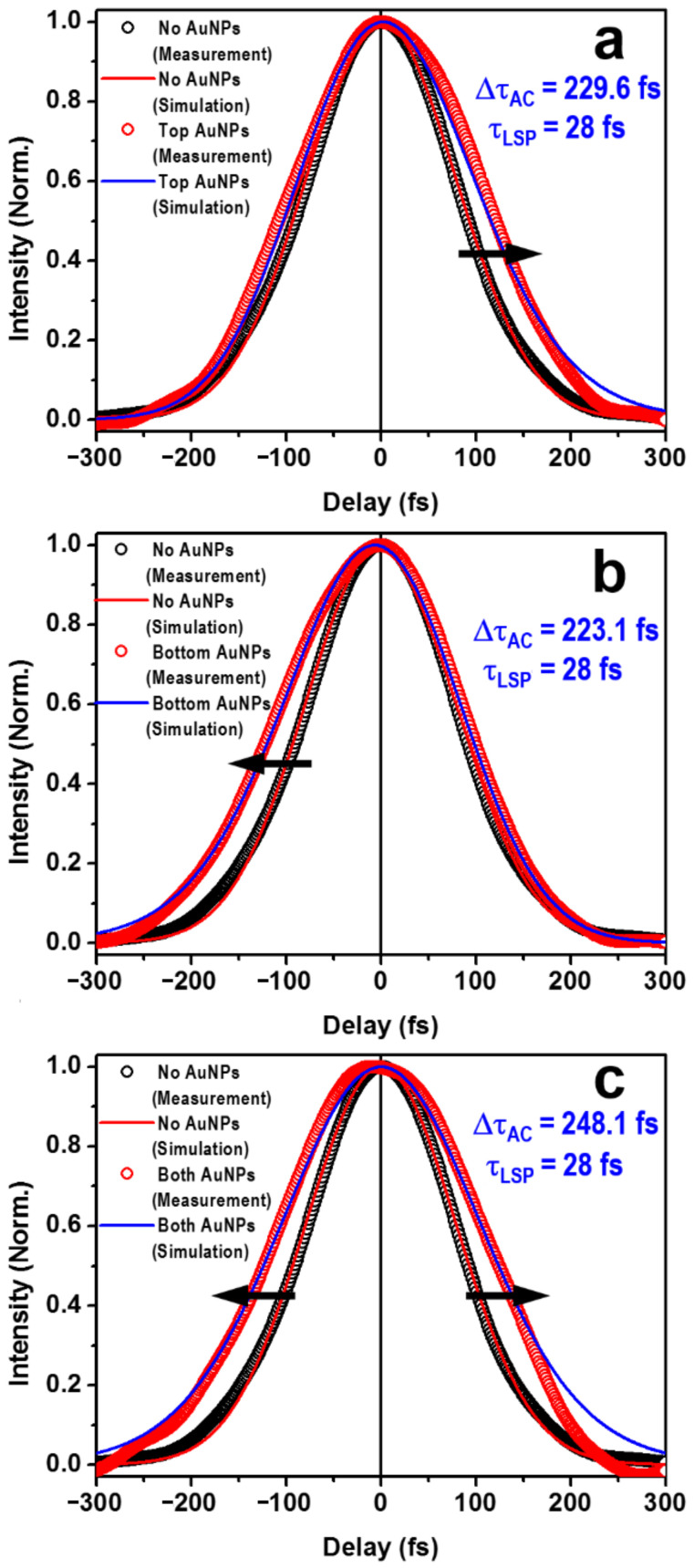
Autocorrelation measurements on the plasmonic response of the AuNPs (red circles) and their comparison with the autocorrelation curve of the blank glass plates (black circles). The corresponding simulation results are plotted by solid curves in blue and red, respectively. (**a**), (**b**), and (**c**) correspond to the schemes ➋, ➌, and ➍, respectively.

Figure 5 shows the experimental results for the autocorrelation signals (empty red circles), where Figure 5a–c correspond to schemes ➋, ➌, and ➍, respectively. For comparison, the measured autocorrelation trace for scheme ➊ with two blank glass plates is represented by the empty black circles. The solid red curves are the calculated autocorrelation traces using the model in Section 3 for an incident pulse length of *τ_P_* = 130 fs, where its excellent agreement with the measurement verified the preciseness of both the incident pulse length and the modeling result. The blue curves are the simulation results with AuNPs involved in autocorrelation processes. The plasmonic dephasing lifetime was justified by fitting the measured autocorrelation data using our model in (1–6). In Figure 5a, the upper glass plate was coated with AuNPs for the rotator, corresponding to scheme ➋. Apparently, the broadening of the autocorrelation trace was predominantly observed on the falling edge, as highlighted by a rightward black arrow. In contrast, by replacing the lower bland glass plate with AuNPs and leaving the upper one blank, the dominant broadening was observed on the rising edge, as shown in Figure 5b and highlighted by a leftward arrow. Such a dependence of the edge-signal effect on the autocorrelator configuration can be explained by the arrangement of the two glass plates and the rotating direction of the autocorrelator. For instance, in a clockwise rotation from an initial position of *α* = 45°, corresponding to the peak intensity of the autocorrelation signal, the optical path length was increased in the upper plate and reduced in the lower plate with increasing rotation angle (*α*). However, for an anti-clockwise rotation, the optical path length was reduced in the upper plate and increased in the lower plate, with respect to the initial position of *α* = 45°. This process determines on which edge of the autocorrelation trace the broadening effect is observed. On such a basis, the broadening of the autocorrelation signal takes places on both edges of the trace if both the upper and lower glass plates are coated with AuNPs, which was verified in Figure 5c, as indicated by the leftward and rightward arrows.

Apparently, such an edge modulation effect does not influence the measurement of the plasmonic response lifetime. However, this is an important indication of how the AuNPs are managed in the autocorrelator scheme. This phenomenon not only verified the true plasmonic modulation on the optical pulse, but also evidenced the modulation manner featured with the autocorrelator design. Fitting the measurement data using the modeling in Equations (7)–(13), we may precisely determine both the pulse length of the incidence and the plasmonic response lifetime. According to the results in Figure 5, we may justify *τ_P_* = 130 fs and *τ_LSP_* = 28 fs, where perfect agreement between the simulation and measurement results were observed. Thus, we determined a plasmonic dephasing lifetime of 28 fs for the random matrix of AuNPs. Such a dephasing lifetime corresponds to the effective modulation or broadening of the incident light pulse.

## 5. Conclusions

We demonstrated a femtosecond autocorrelation scheme for the measurement of plasmon electronic dephasing lifetimes. A rotator autocorrelator consisting of two cross-stacked glass plates was central to the design, where one or both of the glass plates were coated with randomly distributed AuNPs for plasmonic modulation on the incident light pulse for autocorrelation measurements. Using 130 fs laser pulses at 800 nm, we were able to determine the plasmonic electron dephasing lifetime as about 28 fs, which was resolved by fitting the experimental data using theoretical simulations. The electronic dynamics of localized surface plasmon resonance was modeled by a damped simple harmonic oscillator, which was verified by the excellent agreement between theory and the measurement data. A third-order polynomial relationship was resolved between the plasmon electronic dephasing lifetime and the width of the autocorrelation trace at FWHM. Compared with the femtosecond pump–probe spectroscopy, this method enables extremely simple, low-cost, and efficient characterization of the plasmon electronic dephasing lifetime. In particular, using low-energy femtosecond laser pulses from a mode-locked laser oscillator, we were able to largely reduce the disturbance by the optical thermal effect and more precisely determine the pure electronic dynamics and related processes. This method for determining the electronic dephasing lifetime of localized surface plasmons applies to any metallic nanostructures that can be fabricated on a transparent planar substrate.

## Data Availability

Not applicable.

## References

[1] Hutter E., Fendler J.H. (2004). Exploitation of localized surface plasmon resonance. Adv. Mater..

[2] Kelly K.L., Coronado E., Zhao L.L., Schatz G.C. (2003). The optical properties of metal nanoparticles: The influence of size, shape, and dielectric environment. J. Phys. Chem. B.

[3] Stockman M.I., Faleev S.V., Bergman D.J. (2001). Localization versus delocalization of surface plasmons in nanosystems: Can one state have both characteristics?. Phys. Rev. Lett..

[4] Balitskii O., Mashkov O., Barabash A., Rehm V., Afify H.A., Li N., Hammer M.S., Brabec C.J., Eigen A., Halik M. (2022). Ligand tuning of localized surface plasmon resonances in antimony-doped tin oxide nanocrystals. Nanomaterials.

[5] Vasić B., Goran I., Radoš G. (2013). Localized surface plasmon resonances in graphene ribbon arrays for sensing of dielectric environment at infrared frequencies. J. Appl. Phys..

[6] Zhang J., Zhou R., Minamimoto H., Yasuda S., Murakoshi K. (2019). Nonzero wavevector excitation of graphene by localized surface plasmons. Nano Lett..

[7] Alqanoo A.A., Ahmed N.M., Hashim M., Almessiere M.A., Taya S.A., Zyoud S.H. (2022). Silver nanowires assisted porous silicon for high photodetector sensitivity using surface plasmonic phenomena. Sens. Actuat. A-Phys..

[8] Wu J.L., Chen F., Hsiao Y., Chien F., Chen P., Kuo C., Huang M.H., Hsu C. (2011). Surface plasmonic effects of metallic nanoparticles on the performance of polymer bulk heterojunction solar cells. ACS Nano.

[9] Lee Y.H., Kim D.H., Yoo K.H., Kim T.W. (2014). Efficiency enhancement of organic light-emitting devices due to the localized surface plasmonic resonant effect of Au nanoparticles embedded in ZnO nanoparticles. Appl. Phys. Lett..

[10] Tang M., Zhu W., Sun L., Yu J., Qian B., Xiao T. (2015). Localized surface plasmons enhanced color conversion efficiency in organic light-emitting device with surface color conversion layer. Synthetic Met..

[11] Long L., He D., Bao W., Feng M., Zhang P., Zhang D., Chen S. (2017). Localized surface plasmon resonance improved lasing performance of Ag nanoparticles/organic dye random laser. J. Alloys Compd..

[12] Sidiropoulos T.P.H., Röder R., Geburt S., Hess O., Maier S.A., Ronning C., Oulton R.F. (2014). Ultrafast plasmonic nanowire lasers near the surface plasmon frequency. Nat. Phys..

[13] Chen H., Wang X., Zhang J., Rao X., Yang H., Qi Y., Tang C. (2023). Theoretical study of surface plasmonic refractive index sensing based on gold nano-cross array and gold nanofilm. Phys. B Condens. Matter.

[14] Wang Y., Ao S., Yang F., Zhang Z., Zhao Y. (2022). Coupling between surface plasmon modes of single-layer complex silver nanohole arrays and enhancing index sensing. ACS Appl. Nano Mater..

[15] Luo X., Qiao L., Xia Z., Yu J., Wang X., Huang J., Shu C., Wu C., He Y. (2022). Shape-and Size-Dependent Refractive Index Sensing and SERS Performance of Gold Nanoplates. Langmuir.

[16] Hu H., Lu X., Chen K., Yan Z., Cai P., Tang C. (2022). Plasmonic Fano-type nanocavity for double resonance enhanced SERS and optical sensing. Opt. Commun..

[17] Zhu Z., Yuan J., Jiang L. (2020). Multifunctional and multichannel all-optical logic gates based on the in-plane coherent control of localized surface plasmons. Opt. Lett..

[18] Sahu P.P. (2023). Fundamental optical logic gate operations using optically controlled two surfaces plasmonic polariton mode coupler based on graphene clad waveguide. Opt. Eng..

[19] Wang Y., Zhang X.P. (2019). Ultrafast optical switching based on mutually enhanced resonance modes in gold nanowire gratings. Nanoscale.

[20] Lin Y.H., Zhang X.P. (2017). Ultrafast multipolar plasmon for unidirectional optical switching in a hemisphere-nanoshell array. Adv. Opt. Mater..

[21] Zhang X.P., Yang J.H. (2019). Ultrafast plasmonic optical switching structures and devices. Front. Phys..

[22] Zhang X.P., He J.F., Wang Y.M., Liu F.F. (2016). Terahertz beat oscillation of plasmonic electrons interacting with femtosecond light pulses. Sci. Rep..

[23] Anderson A., Deryckx K.S., Xu X.G., Steinmeyer G., Raschke M.B. (2010). Few-femtosecond plasmon dephasing of a single metallic nanostructure from optical response function reconstruction by interferometric frequency resolved optical gating. Nano Lett..

[24] Hubenthal F. (2007). Ultrafast dephasing time of localized surface plasmon polariton resonance and the involved damping mechanisms in colloidal gold nanoparticles. Prog. Surf. Sci..

[25] Zeng P., Cadusch J., Chakraborty D., Smith T.A., Roberts A., Sader J.E., Davis T.J., Gómez D.E. (2016). Photoinduced electron transfer in the strong coupling regime: Waveguide-plasmon polaritons. Nano Lett..

[26] Lamprecht B., Leitner A., Aussenegg F.R. (1999). SHG studies of plasmon dephasing in nanoparticles. Appl. Phys. B.

[27] Lamprecht B., Leitner A., Leitner A., Aussenegg F.R. (1999). Resonant and off-resonant light-driven plasmons in metal nanoparticles studied by femtosecond-resolution third-harmonic generation. Phys. Rev. Lett..

[28] Bosbach J., Hendrich C., Stietz F., Vartanyan T., Träger F. (2002). Ultrafast dephasing of surface plasmon excitation in silver nanoparticles: Influence of particle size, shape, and chemical surrounding. Phys. Rev. Lett..

[29] Sönnichsen C., Franzl T., Wilk T., Plessen G.V., Feldmann J., Wilson O., Mulvaney P. (2002). Drastic reduction of plasmon damping in gold nanorods. Phys. Rev. Lett..

[30] Zentgraf T., Christ A., Kuhl J., Giessen H. (2004). Tailoring the ultrafast dephasing of quasiparticles in metallic photonic crystals. Phys. Rev. Lett..

[31] Hubenthal F., Hendrich C., Träger F. (2010). Damping of the localized surface plasmon polariton resonance of gold nanoparticles. Appl. Phys. B.

[32] Lin W., Shi Y., Yang X., Li J., Cao E., Xu X., Pullerits T., Liang W., Sun M. (2017). Physical mechanism on exciton-plasmon coupling revealed by femtosecond pump-probe transient absorption spectroscopy. Mater. Today Phys..

[33] Aeschlimann M., Brixner T., Fischer A., Hensen M., Huber B., Kilbane D., Kramer C., Pfeiffer W., Piecuch M., Thielen P. (2016). Determination of local optical response functions of nanostructures with increasing complexity by using single and coupled Lorentzian oscillator models. Appl. Phys. B.

